# New Volvovirus Isolates from *Acheta domesticus* (Japan) and *Gryllus assimilis* (United States)

**DOI:** 10.1128/genomeA.00328-13

**Published:** 2013-06-06

**Authors:** Hanh T. Pham, Hajime Iwao, Max Bergoin, Peter Tijssen

**Affiliations:** INRS-Institut Armand-Frappier, Laval, QC, Canadaa; Niigata City Aquarium, Nishifunami, Niigata, Japanb

## Abstract

A novel circular single-stranded DNA (ssDNA) virus, volvovirus, from the house cricket has been described recently. Here, we report the isolation of volvoviruses from *Acheta domesticus* in Japan and *Gryllus assimilis* in the United States. These *Acheta domesticus* volvovirus (AdVVV) isolates have genomes of 2,517 and 2,516 nucleotides (nt) and 4 large open reading frames (ORFs).

## GENOME ANNOUNCEMENT

Billions of pet-feeder crickets are produced annually (1, 2). Since September 2009, stocks of the preferred house cricket, *Acheta domesticus*, were decimated due to severe fatal outbreaks caused by a densovirus, the *Acheta domesticus* densovirus (AdDNV) (2, 3). Similarly, in Japan house cricket stocks were decimated since early in the summer of 2009 despite strict import and export regulations. To avoid heavy losses, many cricket producers switched to the Jamaican field cricket, *Gryllus assimilis* (Fabricius) (1).

Some recent samples contained a new single-stranded, circular DNA virus (4), volvovirus or *Acheta domesticus* volvovirus (AdVVV)-IAF, that is not related to cycloviruses (5, 6), circoviruses (7, 8, 9), nanoviruses (10, 11), or geminiviruses (12, 13, 14).

Electron microscopy of diluted extracts from recently obtained dead house crickets from Japan and dead Jamaican field crickets from U.S. producers revealed highly concentrated icosahedral particles of about 18 nm diameter. Virus and nucleic acid were extracted as described previously (4). The nucleic acid was resistant to RNase and restriction enzymes, suggesting a single-stranded DNA (ssDNA) genome. Native viral DNA was used for double-strand DNA synthesis at 30°C by phi29 DNA polymerase (3, 15). Amplified DNA was digested with MboI or EcoRI, cloned into the BamHI or EcoRI sites of the pBluescriptSK(-) vector, and sequenced by Sanger’s method and primer walking as described before (4, 16). The sequences were assembled by the CAP3 program (17) and generated a 2,517-nucleotide (nt) sequence for the Japanese isolate (AdVVV-Japan) and a 2,516-nt sequence for the *Gryllus assimilis* isolate (AdVVV-Ga). Both were closely related to AdVVV-IAF (4) and contained a single EcoRI site. PCR using native viral DNA and 2 sets of outward primers (with respect to the EcoRI fragment) and sequencing confirmed the circular nature of the genome. Nucleotide numbering was as for AdVVV-IAF (4).

Among open reading frames (ORFs) coding for >100 amino acids (aa), ORF1 (361 aa, starting at nt 447), putatively coding for the capsid protein (CP), and ORF4 (130 aa, starting at nt 70) were in the sense direction, whereas ORF2 (270 aa, starting at nt 2445 for AdVVV-Japan but at nt 2444 for AdVVV-Ga) and the overlapping ORF3 (207 aa, starting at nt 2393 for AdVVV-Japan but at 2392 for AdVVV-Ga) were in the antisense direction. BLASTp revealed a maximum identity of about 30% between ORF2 and rep proteins of circoviruses and cycloviruses, with coverage of ~85%. Compared to the original AdVVV-IAF isolate, AdVVV-Japan contained 22 substitutions of which none were in the overlapping ORF2 and ORF3, and only one was in the intergenic regions. Surprisingly, the bulk of substitutions were in the putative CP (18, of which 14 were nonsynonymous or 4% of protein sequence), whereas the putative ORF4 protein contained 3 nonsynonymous substitutions. Also striking was the high arginine content of the N terminus of the CP. The AdVVV-Ga isolate, compared to AdVVV-IAF, contained 4 nonsynonymous substitutions in the capsid protein also found in the Japanese isolate and one additional nonsynonymous substitution. It also had a deleted T at position 1620 in the intergenic region.

In conclusion, volvoviruses seem to be widespread and to infect several species of crickets.

### Nucleotide sequence accession numbers. 

The GenBank accession number of AdVVV-Ga is KC794539 and that of AdVVV-Japan is KC794540.
